# Assessment of organ dose reduction and secondary cancer risk associated with the use of proton beam therapy and intensity modulated radiation therapy in treatment of neuroblastomas

**DOI:** 10.1186/1748-717X-8-255

**Published:** 2013-11-01

**Authors:** Hiroshi Fuji, Uwe Schneider, Yuji Ishida, Masahiro Konno, Haruo Yamashita, Yuki Kase, Shigeyuki Murayama, Tsuyoshi Onoe, Hirofumi Ogawa, Hideyuki Harada, Hirofumi Asakura, Tetsuo Nishimura

**Affiliations:** 1 Division of Proton Therapy, Shizuoka Cancer Center Hospital, 1007 Shimonagakubo, Nagaizumi, Shizuoka 411-8777, Japan; 2 Division of Pediatric Oncology, Shizuoka Cancer Center Hospital, 1007 Shimonagakubo, Nagaizumi, Shizuoka 411-8777, Japan; 3 Division of Radiation Oncology, Shizuoka Cancer Center Hospital, 1007 Shimonagakubo, Nagaizumi, Shizuoka 411-8777, Japan; 4 Science Faculty, University of Zurich and Institute for Radiotherapy, Radiotherapy Hirslanden AG, Aarau, Switzerland

**Keywords:** Proton beam therapy, Secondary cancer, Neuroblastoma

## Abstract

**Background:**

To compare proton beam therapy (PBT) and intensity-modulated radiation therapy (IMRT) with conformal radiation therapy (CRT) in terms of their organ doses and ability to cause secondary cancer in normal organs.

**Methods:**

Five patients (median age, 4 years; range, 2–11 years) who underwent PBT for retroperitoneal neuroblastoma were selected for treatment planning simulation. Four patients had stage 4 tumors and one had stage 2A tumor, according to the International Neuroblastoma Staging System. Two patients received 36 Gy, two received 21.6 Gy, and one received 41.4 Gy of radiation. The volume structures of these patients were used for simulations of CRT and IMRT treatment. Dose–volume analyses of liver, stomach, colon, small intestine, pancreas, and bone were performed for the simulations. Secondary cancer risks in these organs were calculated using the organ equivalent dose (OED) model, which took into account the rates of cell killing, repopulation, and the neutron dose from the treatment machine.

**Results:**

In all evaluated organs, the mean dose in PBT was 20–80% of that in CRT. IMRT also showed lower mean doses than CRT for two organs (20% and 65%), but higher mean doses for the other four organs (110–120%). The risk of secondary cancer in PBT was 24–83% of that in CRT for five organs, but 121% of that in CRT for pancreas. The risk of secondary cancer in IMRT was equal to or higher than CRT for four organs (range 100–124%).

**Conclusion:**

Low radiation doses in normal organs are more frequently observed in PBT than in IMRT. Assessments of secondary cancer risk showed that PBT reduces the risk of secondary cancer in most organs, whereas IMRT is associated with a higher risk than CRT.

## Introduction

Neuroblastoma is the most common extracranial solid tumor, accounting for >7% malignancies in patients >15 years. Approximately 40% patients present with retroperitoneal mass involving the adrenal gland or neural ganglion. A multi-disciplinary approach is normally established to treat the disease. Postoperative radiotherapy for the primary site plays a major role in decreasing the recurrence of diseases for advanced stage patients with high-grade histology. However, concerns about late adverse events in irradiated organs remain for survivors of the disease. More than 90% survivors have been reported to suffer from one or more treatment-related event(s) [1].

Although the incidence of secondary cancer in patients with neuroblastoma is not high, it is a profound issue for patients. The reported mortality rate is ten times higher than that of the general population [2]. Therefore, reducing secondary cancer risk from radiotherapy is a major goal of the development of neuroblastoma treatment as well as those of other pediatric cancer [3,4].

Recent advanced techniques of radiotherapy allow the radiation oncologist to optimize the treatment without compromising its effectiveness. The standard conventional radiotherapy (CRT) for retroperitoneal neuroblastoma poses a large dose bath outside the target and deposits high doses to normal organs. Compared with CRT, recent advanced techniques, such as intensity-modulated radiotherapy (IMRT) and proton beam therapy (PBT), are known to provide superior conformal dose distribution for large, irregularly shaped tumors. There are a few reports that characterize these treatments for neuroblastoma [5-9]. However, the dose volume profiles of normal organs in these reports were not consistent or large enough to generalize the technique replacing conventional radiotherapy. Furthermore, we have little knowledge to interpret the superiorities of their dose volume profiles with respect to clinical advantages because the doses of analyzed organs were lower than the ablative dose for the organs, in most cases regardless of treatment techniques.

Recently, surveys of cancer survivors and analysis of their treatment have enabled estimation of the risk of secondary cancer by radiotherapy [10-14]. The method consists of unique dose–response curves and effective tools for estimating the clinical significance of a wide range of doses. The model also includes estimation of the neutron dose [15].

The purpose of this study was to compare the dose–volume parameters in several organs from PBT, IMRT, and CRT. Based on the dose volume analysis, an estimation of secondary cancer risks in these organs was performed using the organ equivalent dose (OED) model, which took into account the rates of cell killing, repopulation, and the neutron dose from the treatment machine.

## Patients and methods

This study was based on treatment planning data for five patients (median age, 4 years; range, 2–11 years) who underwent PBT for truncal neuroblastoma at our hospital from July 2003 to October 2010. According to the International Neuroblastoma Staging System, four children were classified as class IV and one as class II. The tumor was eliminated in three patients, but microscopic residual disease was reported. The rest of two patients were determined to be inoperable after chemotherapy and one of them were recurrent disease. The targeted areas of these patients was bilaterally extended in two patients and unilaterally localized in three. Of the patients with unilateral lesions, two were left-sided one was right-sided. Characteristics of the patients were shown in Table 1. This study was approved by the Institutional Review Board of our institute, and informed consent was waived because of the retrospective nature of the study.

**Table 1:** Patient characteristics

**Case no.**	**Age (y) /Gender**	**Site**	**INSS**	**N-myc amp.**	**Risk**	**Residual tumor status**	**CTV** **(ml)**	**Prescribed dose [Gy (RBE)]**
1	4/F	Rt. Retroperitoneum	4	-	High	Inoperable	147	41.4
2	11/M	Upper abdomen Mediastinum	4	-	High	Recurrence	105	36
3	5/M	Upper abdomen Mediastinum	4	-	High	Complete resection	172	21.6
4	2/F	Lt. Adrenal	4	48	High	Gross total resection	464	21.6
5	2/F	Lt. Adrenal	2A	217	High	Subtotal resection	108	36

### Proton beam therapy (PBT)

All patients underwent PBT to the tumor bed and tumor itself, if it existed. The proton beam was delivered using a rotational gantry system for two to five directions of the beam. The patients were immobilized by vacuum fixation bag during treatment sessions. Orthogonal fluoroscopy was used before every treatment session to verify beam localization. Radiation doses of 21–41 Gy were delivered, depending on the clinical stage, status of remaining tumor, and previous treatment. Taking into account the relative biological effect of a proton beam, the dose was reported in gray, Gy (relative biological effectiveness, RBE), which is equivalent to the physical dose in Gy multiplied by 1.1.

Computed tomography (CT) dedicated to treatment planning was obtained before treatment and fused with CT images and magnetic resonance images obtained before surgery. The combination of images allowed specification of the gross target volume (GTV), which consisted of the tumor bed and areas of residual disease. ^131^Iodine metaiodobenzylguanidine (MIBG) scintigraphy was performed to define the residual tumor in some cases. The clinical target volume (CTV) was created by expanding the GTV by 1–2 cm. If residual disease was subjected to boost irradiation, a CTV for the boost was created for the residual disease. Margins of 2–3 mm were added to the CTVs for initial irradiation and boosting irradiation to obtain the planning target volume.

The parameters of objectives are shown in Table 2. We planned to deliver a prescribed dose to the isocenter of the CTV. The minimum and maximum doses in the CTV were determined to be 80% and 110% of the prescribed dose, respectively. Among the parameters for deposited organ dose, which were prioritized for optimization of the beam configuration, the kidney dose was limited to a designated constraint dose: 12 Gy of mean dose.

**Table 2:** Dose profiles of each organs

		**Dose (Gy)**			**Fraction of cases decrease with respect to CRT (%)**	
		**CRT**	**IMRT**	**PBT**	**IMRT**	**PBT**
Liver	Mean	14.2	13.3	6.3	60	100
	D _5%_	32.1	25.3	22.8	100	100
	D_50%_	10.0	12.8	3.5	40	100
	D_90%_	2.8	4.1	0.2	0	100
Stomach	Mean	20.3	21.5	11.8	60	80
	D _5%_	32.9	31.7	20.4	80	100
	D_50%_	17.7	20.7	5.0	60	100
	D_90%_	10.5	13.5	2.2	40	100
Small intestine	Mean	9.4	11.2	3.4	20	100
	D _5%_	31.4	30.6	21.7	60	100
	D_50%_	3.7	7.6	1.4	40	100
	D_90%_	0.7	1.1	0.0	0	100
Bone	Mean	20.5	21.7	16.9	20	80
	D _5%_	32.2	33.0	27.5	0	100
	D_50%_	21.8	24.7	7.7	40	100
	D_90%_	1.3	4.3	0.1	0	100
Pancreas	Mean	28.2	28.2	21.2	60	80
	D _5%_	32.3	33.2	30.6	20	100
	D_50%_	31.5	31.1	20.9	60	80
	D_90%_	17.0	16.8	11.9	40	80
Colon	Mean	9.9	11.2	2.1	60	100
	D _5%_	31.8	26.6	14.0	100	100
	D_50%_	8.5	10.4	0.1	20	100
	D_90%_	0.7	0.9	0.0	0	100

Doses of other organs, including stomach, liver, small intestine, and rectum were also considered in the beam configuration process. Symmetric irradiation of the vertebral body was also taken into account to prevent asymmetric bone growth. Of each vertebral body, the differences in lateral edges of the vertebral body were limited to <4 Gy, 10–20% of prescribed dose. The total of 2–3 ports were used for a patient.

### Planning for simulation and dose comparison

These axial CT data sets with structures, which were used for planning photon beam treatment, were transferred to the Pinnacle (Phillips Medical Systems, Fitchburg, WI) treatment planning system. Identical objectives for dose delivery to target and organs at risk were applied for optimization of the CRT and IMRT treatment plans. The CRT planning was used opposed anterior-posterior and posterior-anterior beams. The IMRT plan was developed using inverse treatment planning. A total of 4–7 beams selected from 18 gantry angles were used with a 0.5 cm × 0.5 cm minimum beam resolution.

### Evaluation of risks of secondary cancer

Secondary cancer risk at the organs of interest was calculated using a mechanistic model of carcinogenesis including cell killing and fractionation effects [10-14].

**Figure 1:**
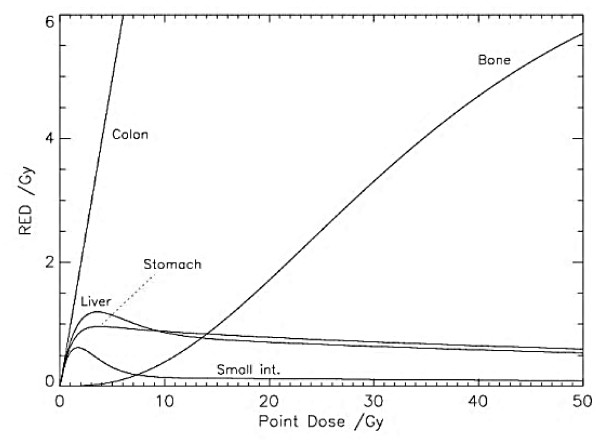
**RED corresponding with organ dose.** The linear–exponential curves may have a plateau or a decline in RED at higher dose.

Dose–volume histograms were computed for liver, stomach, colon, small intestine, pancreas, and bone for all patients and all treatment techniques, using the treatment planning system. The dose–volume histograms were corrected for neutron dose on the basis of neutron dose measurements at the used proton beam line [16]. The dose response relationship for radiation induced cancer, in this work called risk equivalent dose (RED), was taken from [10] for the organs plotted in Figure 1. It is defined as

$$\mathrm{RED}\left(D\right)=\frac{e^{-\alpha^{\prime }D}}{\alpha^{\prime }R}\left(1-2R+R^{2}e^{\alpha^{\prime }D}-{\left(1-R\right)}^{2}e^{-\frac{\alpha^{\prime }R}{1-R}\;D}\right)\;$$

Where *D* is the dose, and *α* and *R* are organ-specific model parameters taken from [10].

The organ equivalent dose (OED), which is a generalized dose average weighted with the dose–response curve for second cancer induction (RED) and thus proportional to the probability for the induction of a malignancy [11], was calculated from the dose–volume histograms:

$$\mathrm{OE}D_{\mathrm{organ}}=\frac{1}{V_{T}}\sum_{i}V\left(D_{i}\right)\mathrm{RED}\left(D_{i}\right)$$

where *V*_*T*_ is the total organ volume and *V(D*_*i*_*)* the dose–volume histogram,. OED values are age independent and can be used to compare different treatment plans with regard to the organ- and plan-specific secondary cancer induction rate.

Secondary cancer risk is highly dependent on age at exposure (*agex*) and increases with decreasing *agex*. Because the patients studied in this work were very young, we decided to compute absolute risk estimates in terms of lifetime attributable risk (LAR), although the LAR presented might be subject to large errors. The LAR was calculated according to Kellerer et al. [15]:

$$\begin{array}{l} \mathrm{LA}R_{\mathrm{organ}}=\int_{\mathrm{agea}-\mathrm{agex}}^{\infty }\mathrm{\beta OE}D_{\mathrm{organ}}\exp\\ \;\times \left(\gamma_{e}\left(\mathrm{agex}-30\right)+1n\left(\frac{\mathrm{agex}}{70}\right)\right)\frac{S\left(\mathrm{agea}\right)}{S\left(\mathrm{agex}\right)}\mathrm{dagex} \end{array}$$

where *S* is the survival function for a standard population, taken from report of Kellerer et al., *β* is the cancer induction rate in percent per person per gray for low dose, and *γ*_*e*_ and *γ*_*a*_ are organ-specific parameters taken from the atomic bomb survivor data [10]. The integration is taken over the attained age *agea*. LAR is then the lifetime attributable cancer risk for a patient treated with the treatment plan/technique in question at *agex*.

## Results

### Dosimetric comparisons

The common prescribed dose to the target and the doses to the organs for all three treatment techniques are shown in Table 2. Among the five organs, the mean dose to the liver in IMRT was lower than that in CRT, although the difference is not statistically significant. Decreases in high dose deposition by IMRT were more prominent than that of low dose deposition. In three of five patients, IMRT reduced D_5%_ of liver, stomach, small intestine, and colon. However, the fraction of patients that showed decreases in D_90%_ in all organs was less than half. The mean doses to stomach, bone, and colon were significantly lower in PBT than in CRT. The decrease in deposited dose by PBT was seen across a wide range (i.e., D_5%_, D_50%_, and D_90%_), except in pancreas.

**Figure 2:**
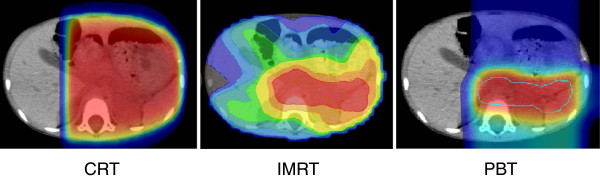
**Dose distributions map of CRT, IMRT and PBT for representative case.** Area irradiated with 95%(red), 70%(yellow), 40%(green) and 10%(blue) of prescribed dose were shown in CT image.

**Figure 3:**
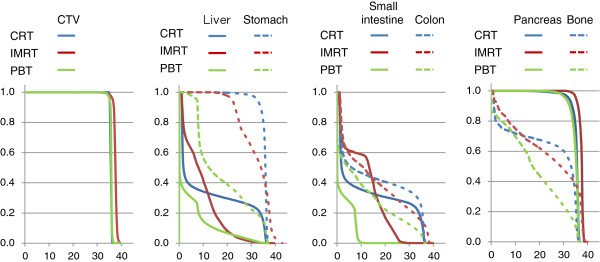
**Dose volume histograms of CRT, IMRT and PBT.** Dose volume histograms were compared among three treatment techniques for CTV and six organs.

The representative dose distribution map and dose volume histograms of the three treatment techniques are shown in Figures 2 and 3. PBT and IMRT had comparable coverage of the target with the prescribed dose and a substantial decrease in volume of dose compared with CRT. IMRT can be characterized as having a larger fraction of organs irradiated at a lower dose compared with CRT. The dose–volume histogram of PBT demonstrated a lower fraction for high dose and low dose deposition.

### Secondary cancer risk

**Figure 4:**
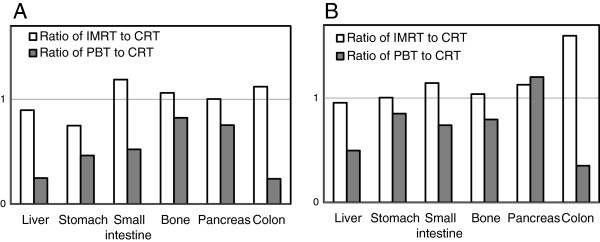
**Mean doses and REDs for evaluated organs.** Ratio of mean organ doses **(A)** and RED **(B)** for IMRT and PBT to those for CRT.

The ratios of secondary cancer risk in IMRT were 0.95 for liver, 1.00 for stomach, 1.03 for bone, 1.24 for colon, 1.14 for pancreas, and 0.81 for the small intestine. The ratios of secondary cancer risk in PBT were 0.49 for liver, 0.83 for stomach, 0.79 for bone, 0.24 for colon, 1.21 for pancreas, and 0.36 for the small intestine. The estimated secondary cancer risks in IMRT were lower than those in CRT for two of the five organs. The estimated secondary cancer risks for the other three organs in IMRT were equal to or higher than those in CRT. The estimated secondary cancer risk in PBT was lower in all organs except pancreas (Figure 4).

## Discussion

Three radiotherapy techniques, CRT, IMRT, and PBT, were compared in terms of delivered dose and estimated risks secondary cancer for normal organs. We found that IMRT has marginal advantages in dose reduction when compared with CRT. The general replacement of CRT with IMRT in the treatment of neuroblastoma is controversial. Paulino reported a substantial variation in organ–dose reduction effect with IMRT in neuroblastoma treatment [10]. Their analysis demonstrated that IMRT increased the dose to normal organs in cases with extending disease or with specific beam configurations. The recent report comparing tomotherapy and 3D CRT also demonstrated inconsistent benefits of IMRT [6]. In their analysis, the volume irradiated at high dose is inclined to be low in IMRT, but the volume irradiated at a low dose in IMRT is larger than that in CRT. This propensity was also observed in the current study.

Although there are patients who derive less advantage from IMRT in normal organ dose reduction, others patients derive apparent benefit from IMRT after prior simulation to select the appropriate treatment technique. The priority of adverse event reduction and the dose-volume parameter to limit the adverse event among variety of organs are uncertain. When the priority of dose-volume parameter are established, it becomes clear that the flexibility of dose delivery in IMRT makes it more effective.

We found that dose to organs in PBT was lower than those deposited by CRT. The superiority of PBT in neuroblastoma treatment compared with CRT was reported in 2001 [7]. The results of the current study, which included patients of varying size and sites, emphasize the usefulness of an early simulation study.

In contrast to IMRT, the reduction of dose to normal organs in PBT is observed at a wide range of dose. Therefore, dose reduction in PBT expected to be robust compared with IMRT.

Comparing dose reduction between IMRT and PBT is the theme of recent focus. Hattangi reported that PBT decreased the dose to organs including stomach and liver compared with IMRT [5]. The current study confirmed the decrease in deposited dose to liver and stomach, and showed that the colon and small intestine also showed a decrease in deposited dose in PBT compared with IMRT. Comparing several dose levels in both PBT and IMRT, PBT showed more apparent decreases in the low and middle dose levels. Therefore, the dose reduction of PBT is expected to be robust compared with IMRT.

Among the five patients in the current study, three cases extended to the retroperitoneum and the clinical target volume of these cases included the pancreas. The organ involved in the designated CTV cannot avoid irradiation, even with a conformal radiotherapy technique such as IMRT or PBT.

We introduced the secondary cancer risk estimation to establish the clinical significance of conformal treatment techniques. For secondary cancer, most clinical data show a linear–exponential relationship. Linear–exponential models were proposed as a result of analysis of cancer incidence in patients who underwent radiotherapy for Hodgkin lymphoma [12]. The concept of including cell sterilization, which occurs at curative doses in radiotherapy, is reasonable and the proximity of the dose range used in this analysis to the therapeutic dose decreases the uncertainties in exploration of their formula. The difference between IMRT and PBT is more apparent in secondary cancer risks, which is obtained by comparing mean doses of organs. The outcome can be used to argue that PBT is more widely applicable.

There are a few reports on estimation of secondary cancer risks using deposited dose to target. Zhang et al. reported the risk of secondary cancer after craniospinal irradiation for medulloblastoma [17]. They report the risk of secondary cancer using a variety of curves for dose–response between deposited dose and excessive relative risk. With variety of formulas for estimating excess absolute risk, including a linear relationship, the ratio of secondary risk was reduced by factor of 0.055–0.36 using PBT compared with using IMRT in medulloblastoma treatment.

Yoon, et al. also simulated craniospinal irradiation with the OED model. They also demonstrated that it decreased the risk of secondary cancer. However, the estimation of cancer risk in their report does not account for differences among organs [18]. The model used in the current study is based on a dedicated linear–exponential curve for each organ, including the parameters extracted from the survey of atomic bomb survivors. Therefore, the estimated numbers are more reasonable with the available data.

The current study demonstrated the discrepancy between the estimated secondary cancer risk of organs and the deposited dose of the organs. The mean dose to the pancreas in IMRT and PBT was lower than that in CRT. However, the estimated risk of secondary cancer in the pancreas with IMRT and PBT is higher than in CRT. Generally, optimization is performed during treatment planning to reduce the dose to normal organs. However, with the OED model, irradiation of organs with a higher dose, over the inflection point, does not necessary increase secondary cancer risk compared with lower dose irradiation. While increasing the dose to a normal organ may favor the reduction of cancer risk under particular conditions, it is thought to increase the risk of functional ablation of the organ [18]. Therefore, we should be careful about increasing the organ dose based on the estimated risks of secondary cancer.

## Conclusions

Low radiation doses in normal organs are more frequently observed in PBT than in IMRT. Assessments of secondary cancer risk showed that PBT reduces the risk of secondary cancer in most organs, whereas IMRT is associated with a slightly larger risk than CRT.

## Competing interests

This research is (partly) supported by the “Funding Program for World-Leading Innovative R&D on Science and Technology (FIRST Program),” initiated by the Council for Science and Technology Policy (CSTP). The authors declare no competing interest.

## Authors’ contributions

HF, US contributed to the concepts and design of the study. HF, US, MK, HY, YK collected the data and all authors contributed to the interpretation of the data. HF drafted the article. All authors commented on the first draft, revised the manuscript and approved the final version.
